# Evaluation of the impact of treatment with hematopoietic stem cells transplantation (HSCT) on biochemical markers of heart function and novel electrocardiographic markers of repolarization in patients with hematological malignancies

**DOI:** 10.1007/s12032-018-1221-5

**Published:** 2018-10-31

**Authors:** Małgorzata Poręba, Paweł Gać, Lidia Usnarska-Zubkiewicz, Witold Pilecki, Kazimierz Kuliczkowski, Grzegorz Mazur, Marzena Gonerska, Małgorzata Sobieszczańska, Rafał Poręba

**Affiliations:** 1 Department of Pathophysiology, Wroclaw Medical University, Marcinkowskiego 1, 50-368 Wroclaw, Poland; 2 Department of Hygiene, Wroclaw Medical University, Mikulicza-Radeckiego 7, 50-368 Wroclaw, Poland; 3 Department of Hematology, Blood Neoplasms and Bone Marrow Transplantation, Wroclaw Medical University, Pasteur 4, 50-367 Wroclaw, Poland; 4 Department of Internal Medicine, Occupational Diseases and Hypertension, Wroclaw Medical University, Borowska 213, 50-556 Wroclaw, Poland

**Keywords:** High-dose chemotherapy, Hematopoietic stem cell transplantation, Natriuretic peptide type B, Troponin, Repolarization markers

## Abstract

High-dose chemotherapy (HDC) followed by stem cell transplantation (HSCT) is a well-established method in patients with hematological malignancies, and for last few years, many efforts have been made to estimate short- and long-term efficacy of this method, as well as early and late complications. The present study concentrates on cardiotoxic effects, mainly early changes using biochemical markers such as N-terminal natriuretic peptide type B (NT-proBNP) and cardiac troponins (cTn). Simultaneously, the analysis of 12-lead ECG was done before and after the procedure in which the novel repolarization markers: *T*_p–e_ and *T*_p–e_/QT ratio were measured, together with standard markers: QT, QTc. It was found that NT-pro BNP was significantly increased after HSCT in comparison to results before it, and no significant changes were present in Troponin levels. Simultaneously, *T*_p–e_ interval and *T*_p–e_/QT ratio were significantly higher after HSCT. The use of cyclophosphamide, advanced age, and higher level of blood cholesterol concentration were risk factors for the increase in NT-proBNP and treatment with cyclophosphamide as well as fludarabine and higher creatinine levels were risk factors for the increase in *T*_p–e_/QT ratio. In conclusion, in the early term evaluation after HSCT in patients with no previously diagnosed heart disease, the mild changes in markers of heart overload and repolarization were noted. The observations suggest that in all patients undergoing HSCT, even the ones without pre-existing cardiovascular disease, the evaluation, and monitoring of heart function should be considered.

## Introduction

The problem of cardiotoxicity of chemotherapy has been known for last decades. However, the influence of the peripheral blood stem cell transplantation with prior high dose chemotherapy is still under study.

A procedure of high-dose chemotherapy (HDC) followed by autologous hematopoietic stem cell transplantation (HSCT) is used to treat numerous diseases. Nowadays, HSCT is a cure for many patients with hematologic malignancies; however, this treatment can also cause early and late complications and still long-term mortality rates remain higher than in the general population [1]. That is why the profound research is conducted in this area, with special attempts to find early markers of probable future complications, which we could use in clinical setting to avoid or fight some of them.

One of the proposed markers to evaluate early cardiotoxic effects are troponin and natriuretic peptides, that is mainly B-type natriuretic peptide (BNP). N-terminal pro-B-type natriuretic peptide (NT-proBNP) and troponin are known biochemical markers, in diagnosis of the heart failure and myocardial infarction, respectively. NT-pro-BNP is secreted by myocardial cells in response to increased pressure and volume and is used to identify left ventricular dysfunction; however, there are more data on supporting the role in predicting future cardiac events especially in coronary artery disease, and new data are being gathered as for the screening tool for left ventricular dysfunction in different populations [2–5].

Roziakova et al. showed that the increase in biomarkers, that is NT-proBNP and hs-cTnT (high-sensitive cardiac troponin T), especially at day 14 after HSCT predicts the future risk of developing cardiac events during next 6 months [6]. In different cancer therapies cardiac biomarkers, such as troponin I and NT-proBNP were checked as good indicators of susceptibility to cardiac toxicity, however, in some cases, they are better investigated in treatment of specific type of chemotherapy such as trastuzumab or tysosine kinase inhibitors or mToR inhibitors (sirolimus, temsirolimus) [7].

It was found that novel electrocardiographic markers connected with dispersion of repolarization such as *T*_p–e_ interval, *T*_p–e_/QT, and *T*_p–e_/QTc ratio can be suitable for prediction of ventricular arrhythmias and mortality [8–10]. Especially, *T*_p–e_ interval as independent of heart rate may be a valuable tool in several diseases including Bruggada syndrome, hypertrophic cardiomyopathy, and in patients with myocardial infarction [10–12].

Up till now, there were rather few reports on the influence of high-dose chemotherapy and HSCT carried out in patients with hematological malignancies on electrocardiographic repolarization markers, that is mainly QTc was under study, indicating that long QT may be a risk factor of non-relapse mortality or heart failure incidence [13, 14].

The aim of the study was to evaluate the analysis of blood concentrations of N-terminal natriuretic peptide type B and cardiac troponin as well as novel electrocardiographic markers of repolarization in patients with hematological malignancies undergoing HSCT with high-dose chemotherapy in early period after the procedure.

## Materials and methods

### Study group

A study group of patients was selected from the candidates to high-dose chemotheraphy with consecutive HSCT, included to the project entitled: “The evaluation of the early cardiotoxicity of high-dose chemotherapy and hematopoietic stem cells transplantation in patients with blood neoplasms”. Inclusion and exclusion criteria and the characteristics of the population participating in the project were presented in a the former publication of the authors [15].

The final study group consisted of 48 patients with blood neoplasms undergoing HSCT with prior HDC procedure in accordance with current guidelines. We excluded from the study group all patients with the previously diagnosed cardiovascular diseases and in exclusion criteria were arterial hypertension, heart failure, diabetes, coronary artery disease, and stroke in medical history. In 28 patients, autologous HSCT was done and in the rest 20 patients—allogeneic HSCT. Type of HDC was appropriate to the diagnosed malignancy, and chemotherapy doses were calculated based on patient’s body weight or body surface area. Clinical characteristics of the study group and results of laboratory tests are presented in Tables 1 and 2. Data on HDC and total body irradiation before HSCT are shown in Table 3.

**Table 1:** Clinical characteristics of the study group

Age (years)	42.23 ± 12.83
Gender (%/*n*)	
Men	56.25/27
Women	43.75/21
Blood neoplasms (%/*n*)	
Acute myeloid leukemia—AML	29.17/14
Multiple myeloma—MM	20.83/10
Hodgkin’s lymphoma—HD	16.67/8
Acute lymphoblastic leukemia—ALL	14.58/7
Non-Hodgkin’s lymphoma—NHL	12.50/6
Chronic myeloid leukemia—CML	6.25/3

**Table 2:** Laboratory characteristics of patients before HSCT

RBC (mln/mL)	3.85 ± 0.50
Hematocrit (%)	35.97 ± 4.54
Hemoglobin (g/dL)	11.75 ± 2.15
WBC (× 10^2^/L)	5.94 ± 2.98
Platelet count (× 10^2^/L)	157.61 ± 90.61
Sodium (mmol/L)	142.07 ± 2.52
Potassium (mmol/L)	4.10 ± 0.38
Prothrombin activity (%)	93.77 ± 11.50
Fibrinogen (mg/dL)	390.00 ± 70.79
APTT (s)	33.77 ± 5.47
Urea (mg/dL)	31.86 ± 11.26
Creatinine (mg/dL)	0.89 ± 0.28
Uric acid (mg/dL)	5.14 ± 1.36
Glucose (mg/dL)	95.15 ± 21.93
Serum protein (g/dL)	6.73 ± 0.65
Albumin (g/dL)	4.20 ± 0.35
Total cholesterol (mg/dL)	190.54 ± 39.45
Triglicerydy (mg/dL)	128.67 ± 46.50
Aspartate aminotransferase (U/L)	20.00 ± 7.91
Alanine aminotrensferase (U/L)	28.09 ± 22.96
Lactate dehydrogenase (U/L)	178.10 ± 28.92
Total bilirubin (mg/dL)	0.78 ± 0.63
C-reactive protein (mg/L)	4.41 ± 6.32

**Table 3:** Cytostatics used in high dose chemotherapy (HDC) and total body irradiation (TBI) before HSCT

Cytostatic (%/*n*)	100.00/48
Melphalan	62.50/30
Carmustine—BCNU	31.25/15
Etoposide	31.25/15
Cytarabine—Ara-C	29.17/14
Busulfan	29.17/14
Cyclophosphamide	25.00/12
Fludarabine	12.50/6
Total body irradiation (%/*n*)	22.92/11

### Methods

In all patients enrolled in the study, cardiac biomarkers were measured in blood, that is NT-proBNP and TnT (Troponin) as well as 12-lead electrocardiography (ECG) was performed; all tests we done twice. First test was carried out prior to the HSCT procedure (test A), and the second—after the whole procedure of HSCT (test B). The precise time of test B was dependent on the patient’s condition, mainly risk of severe infection, and the level of granulocytes, and ranged between 13 and 41 days and mean time between test A and test B was 25.86 ± 8.75 days.

### NT-proBNP and troponine T measurements

From each patient, a sample of heparinized venous whole blood was taken twice for troponin T and NT-proBNP and analyzed with the use of the Cardiac Reader (Roche Diagnostics, Indianapolis). For troponin T measurement range was 0, 1–2 ng ng/mL. Values above or below this range are displayed as “HI” for high or “LO” for low, and for NT-proBNP, measurement range was: 6–3000 pg/mL and values above or below this range are displayed as “HI” for high or “LO” for low, respectively.

### Novel electrocardiographic markers of repolarization

In each patient, standard 12-lead surface ECG was performed at rest in supine position with a paper speed of 50 mm/s and a voltage of 10 mm/mV (Aspel, Zabierzów, Poland). Duration of QRS, QT interval, and *T*_p–e_ interval were measured manually in the precordial leads and checked with the caliper and *T*_p–e_/QT ratio was calculated from these measurements. *T*_p–e_ interval was defined as the time from the peak to the end of T wave and it is considered to represent transmural dispersion index of the ventricular repolarization [9, 10, 16]. The QT interval was corrected for heart rate using Bazett formula (QTc). The end of the T wave was defined as the intersection of the tangent to the downslope of T wave and the isoelectric line.

### Ethical approval

The research has been conducted in compliance with the principles of Good Clinical Practice and Declaration of Helsinki, on the basis of the consent from the Wroclaw Medical University Bioethical Committee. The written consent has been obtained from all the patients taking part in the research. All data collected from the patients were anonymized.

### Statistical analysis

Statistic analysis was conducted using the STATISTICA 12 software (StatSoft Polska). For the quantitative variables, arithmetic means and standard deviations of estimated parameters were calculated. Distribution of variables was examined using Lilliefors and *W*-Shapiro–Wilk tests. For the dependent quantitative variables of the normal distribution, the t test for linked variables was used. In case of quantitative dependent variables showing the distribution distinct from normal, the pair sequence test of Wilcoxon was applied. Results for qualitative variables were expressed in a form of percentages. For dependent qualitative variables, statistical analysis involved the test of McNemar or the Cochran test. In order to define the relationships between variables, analysis of multivariable regression was performed. Parameters of the model obtained in regression analysis were estimated using the technique of least squares. Results at the level of *p* < 0.05 were assumed to be of statistical significance.

## Results

On analysis of biochemical markers of heart function, no increase in Troponin T levels was found before and after HSCT. However, NT-proBNP concentration was statistically significantly higher after transplantation in comparison with the results before the procedure. In electrocardiographic parameters of repolarization, no differences were found in QTc before and after the transplantation, but *T*_p–e_ interval and *T*_p–e_/QT ratio were significantly higher after HSCT than before the procedure. The results of biochemical of as well as electrocardiographic parameters are presented in Table 4.

**Table 4:** Biochemical and electrocardiographic parameters in the study group in test A and B

	Test A	Test B	*p*
Troponin T (µg/L)	0.00 ± 0.00	0.00 ± 0.00	ns
Brain natriuretic peptide—BNP (pg/mL)	174.45 ± 145.17	223.19 ± 136.48	*p* < 0.05
QTc interval (ms)	401.15 ± 24.16	410.26 ± 28.45	ns
*T*_p–e_ interval (ms)	79.18 ± 12.24	92.15 ± 11.58	*p* < 0.05
*T*_p–e_/QT ratio	0.19 ± 0.09	0.24 ± 0.10	*p* < 0.05

Multivariate backward regression analysis enabled to assess the following models:

$$\Delta {\text{BNP }}={\text{ }}0.18\;{\text{ age }}+{\text{ }}4.64\;{\text{ cyclophosphamide }}+{\text{ }}0.05\;{\text{ total }}\;{\text{cholesterol}};$$

$$\Delta {{\text{T}}_{{\text{p}} - {\text{e}}}}/{\text{QT }}\;{\text{ratio }}={\text{ }}0.21{\text{ }}\;{\text{age }}+{\text{ }}2.63\;{\text{ fludarabine }}+{\text{ }}6.80\;{\text{ cyclophosphamide }}+{\text{ }}3.15{\text{ }}\;{\text{creatinine}}.$$

The obtained models show that in the group of patients with hematologic malignancies undergoing HDC in the course of HSCT the use of cyclophosphamide, advanced age, and higher initial blood level of total cholesterol represent the independent risk factors for the increase in NT-proBNP concentration (displayed as the increased difference between test A and test B). Moreover, it was shown that in the study group, treatment with cyclophosphamide and fludarabine as well as the higher creatinine blood concentration were the independent risk factors for the increased *T*_p–e_/QT ratio (expressed as higher difference between test B and test A). The results of estimations for the models obtained in regression analysis are presented in Table 5.

**Table 5:** Results of estimation for the final models obtained in the multivariable step-wise backward regression analysis

Model for ΔBNP				
	Age (years)	Cyclophosphamide	Total cholesterol (mg/dL)	
Regression coefficient	0.179	4.644	0.047	
SEM of Rc	0.105	1.982	0.013	
*p* value	0.028	0.046	0.026	
*p* value for the model	*p* < 0.011			

Model dla Δ*T*_p–e_/QT ratio				
	Age (years)	Fludarabine	Cyclophosphamide	Creatinine (mg/dL)
Regression coefficient	0.215	2.629	6.804	3.152
SEM of Rc	0.117	1.317	2.673	1.455
*p* value	0.029	0.045	0.016	0.038
*p* value for the model	*p* < 0.003			

*ΔBNP* increase in BNP in the test B in comparison with the test A, *ΔT*_p–e_/QT *ratio* increase in *T*_p–e_/QT ratio in the test B in comparison with the test A, *Cyclophosphamide, Fludarabine* nominal variables, where 1: yes, 2: no, *SEM of Rc* standard error of the mean of regression coefficient

## Discussion

It is known that HSCT recipients are at 2–4-fold higher risk of death due to heart complications when compared with general population [17–19]. Thus, the problem of heart function monitoring in this group of patients seems to be very important, in short and also longer period after transplantation. The early, immediate heart failure after HSCT is a quite well defined status, when late complications, that is, after year of HSCT are more poorly investigated. In potential risk factors of developing late heart failure, there are included, similarly as for the early complications such factors are as: pretransplantation exposure to anthracyclines, the use of alkylating agents such as cyclophosphamide, quite often used during HSCT procedures, and mediastine irradiation, compounded by high-dose cyclophosphamide, as well as total body irradiation (TBI) at the time of HSCT [20–23].

NT-proBNP a known marker of heart failure released in the ventricles in response to pressure overload or stretching of myocytes are also used together with troponin concentration, a marker of myocardial injury, in diagnosis of cardiotoxity after anti-cancer treatment [7, 24]. They were adopted in evaluation of cardiotoxicity of anthracyclines, cyclophosphamide, and other agents, however, only few reports were describing their role in HSCT [6, 25–27]. There are reports about usefulness of those markers in different situations for example NT-proBNP appeared to be the most sensitive index of myocardial dysfunction and the most powerful prognostic determinant in numerous types of anti-cancer treatment [7, 28, 29]. Up till now, the most profound study on biochemical markers in HSCT presented Roziakowa et al. [6]. Authors in their scientific investigations on biochemical markers in patients undergoing allogeneic HSCT found that NT-proBNP at day 14 after the transplant may be prognostic for developing cardiac events in next future 6 months. Simultaneously, they claimed that patients who presented persistent elevation of NT-proBNP also had increased hs-cTnT (high sensitive cardiac troponin) and that in some patients the increase in NT-proBNP may be extended even to 180 days after HSCT [6].

In our results, we observed no elevations of troponin level in patients undergoing HSCT, and we suggest that it was a result of the selection of the study group as we excluded all patients with the previously diagnosed heart disease and other cardiovascular status that may increase the potential risk of developing cardiac complications. As to NT-pro BNP concentration, we have shown the elevation after transplantation in comparison with initial level, noted before the procedure, that is, the second test was done on average on the 25 day after first evaluation. Moreover, in our study, it was disclosed that the use of cyclophosphamide, older age, and higher blood cholesterol concentration were risk factors for the increase in NT-proBNP in the second test after HSCT. In our opinion, it is the next evidence that alkylating agent, namely, cyclophosphamide may have the influence on cardiotoxicity measured by biochemical markers in the setting of stem cell transplantation. In the interesting study of Sandri, authors underlined the usefulness on NT-proBNP after high-dose therapy with cyclophosphamide [29]. Additionally, the two other factors had the impact in our analysis: age and cholesterol level both belonging to cardiovascular risk factors potentially connected with higher atherosclerosis burden and probably, it should be suggested that patients undergoing HSCT in older age and with higher cholesterol concentration will be more prone to develop cardiac side effects [30]. It has been determined by Premstaller et al. that in patients with HSCT, dyslipidemia happens in about 36% of patients who receive autologous transplant and 28% in allogeneic group, respectively [31]. In our study group before HSCT, the mean level of cholesterol was 190.54 ± 39.45 mg/dL, which was not high as we excluded patients with diagnosed previously cardiovascular events and diseases. Thus, our group represented rather population with lower cardiovascular risk as it is known, the borderline high cholesterol level starts from 200 mg/dL according to NCEP ATP3 [32]. However, the observation should be taken into account and determining cholesterol level in panel of biochemical tests before HSCT should always be a standard. Although this is not a recommendation now, the administration of statins in patients with higher blood concentrations of cholesterol before HSCT could be suggested; however, further studies are needed in this field. Eventually, it should be underlined that generally the HSCT procedure caused mild cardiotoxic effect which we have measured by blood NT-proBNP concentration.

Generally, to the proposed diagnostic tools for the detection of cardiotoxicity belong, basically echocardiography, cardiac magnetic resonance, and MUGA (nuclear cardiac imaging) by which the ejection fraction of the left ventricle (LVEF) may be assessed (24). However, there is a discussion in this matter that such proceeding may underestimate minor and early changes which could be seen in other tests [6, 24, 33]. That is why biochemical markers and other indices of cardiotoxicity are within the scope of interest and especially NT-proBNP or BNP were found to be elevated after chemotherapy and in few reports also after HSCT [1, 6, 34–37]. Some controversies were also noted and, finally, the need for long-term recommendations for cardiac monitoring has been suggested in last few years [38].

The another problem discussed in our study was the search for the connection between cardiotoxicity and repolarization markers. Some authors revealed that QTc may be prolonged in patients undergoing HSCT and Akahori discovered that not only the QTc had the tendency to be longer after HSCT, but also it was associated with the higher incidence of heart failure with a predictive value [13, 14]. Additionally, in the study conducted by Nakamae at al., it was disclosed that QTc dispersion could be used as a powerful noninvasive predictor of the development of acute heart failure after HSCT [39]. In recent years, there are more observations that *T*_peak_–*T*_end_ parameter measured from 12-lead ECG, best from precordial leads, represents transmural dispersion of repolarization reflecting changes in spatial dispersion of repolarization, particularly transmural dispersion of repolarization, and it may be connected with arrhythmias [8–10, 40]. It has been shown that *T*_peak_–*T*_end_ parameter is better in predicting TdP, the serious ventricular arrhythmia, in patients with long QT syndrome [41, 42]. Also better than QTc, it predicted sudden cardiac death in case of hypertrophic cardiomyopathy and ventricular tachycardia in high-risk patients with organic heart disease [43, 44]. Although, still this marker needs some more validation, which has been suggested by some authors [1, 3, 14, 43].

In our study, no connection was found between QTc before and after HSCT; however, *T*_p–e_ interval and *T*_p–e_/QT ratio were significantly prolonged after the transplantation in comparison with the test before it, which suggests the impaired dispersion of repolarization. The novel markers of repolarization may be very promising tools for future helping to predict the incidence of heart failure and arrhythmias. It is known that arrhythmias, when they happen, may precede the forthcoming cardiac pathology as cardiomyopathy or heart failure also as a complication due to cardiotoxicity. From this point of view, 24-h Holter monitoring should be recommended routinely at peri-transplant period as we suggested in our previous paper [15]. Generally, arrhythmias occur in patients who had the procedure of HSCT in about 7–9% of patients, ranging from 1 to 27%, depending on the methods of evaluation, especially in subjects with a pre-existing cardiovascular disease or with smoking habit [15, 45–47]. However, in our former study, we commented that there are more electrocardiographic pathologies in such a population of patients, but previous publications concentrated mainly on more severe arrhythmias like ventricular tachycardia or atrial fibrillation. We have found more different pathologies including atrio-ventricular blocks and premature beats and, eventually, we noted various changes increasing in up to 41.07% of patients after HSCT [15].

Limitation of the study may be as usual in this kind of study not numerous number of patients and heterogeneity of the study group. It is, however, due to the specificity of the type of the population undergoing HSCT and organization of the Transplantation Department, and additionally for the fact we specially excluded patients with prior diagnosed cardiovascular disease or status to obtain the initial low-risk population.

## Conclusions

1. In patients with hematologic malignancies undergoing high-dose chemotherapy and HSCT, higher blood concentrations of NT-pro BNP were noted and additionally, higher values of *T*_p–e_ interval and *T*_p–e_/QT ratio in electrocardiographic recording after the procedure in comparison with results obtained before this treatment
2. In a group of patients with blood neoplasms undergoing HSCT the use of cyclophosphamide, advanced age and higher cholesterol level are the independent risk factors for the increase in NT-proBNP level after the procedure and the administration of cyclophosphamide and fludarabine are independent risk factors for the increase in *T*_p–e_/QT ratio in electrocardiographic evaluation after HSCT.
